# Nonlinear Thermomechanical Low-Velocity Impact Behaviors of Geometrically Imperfect GRC Beams

**DOI:** 10.3390/ma17246062

**Published:** 2024-12-11

**Authors:** Tao Zhang, Qiang Li, Jia-Jia Mao, Chunqing Zha

**Affiliations:** 1 Beijing Key Laboratory of High Dynamic Navigation Technology, Beijing Information Science & Technology University, Beijing 100192, China; zt@bistu.edu.cn; 2 Beijing Institute of Control & Electronic Technology, Beijing 100038, China; ts.lee@foxmail.com; 3 Beijing Key Laboratory of Nonlinear Vibrations and Strength of Mechanical Structures, Department of Mechanics, School of Mathematics, Statistics and Mechanics, Beijing University of Technology, Beijing 100124, China; 4 Key Laboratory of Advanced Manufacturing Technology, Beijing University of Technology, Beijing 100124, China

**Keywords:** geometric imperfection, thermo-mechanical load, low-velocity impact, graphene nanoplatelets, nonlinear Hertz contact law

## Abstract

This paper studies the thermomechanical low-velocity impact behaviors of geometrically imperfect nanoplatelet-reinforced composite (GRC) beams considering the von Kármán nonlinear geometric relationship. The graphene nanoplatelets (GPLs) are assumed to have a functionally graded (FG) distribution in the matrix beam along its thickness, following the X-pattern. The Halpin–Tsai model and the rule of mixture are employed to predict the effective Young modulus and other material properties. Dividing the impact process into two stages, the corresponding impact forces are calculated using the modified nonlinear Hertz contact law. The nonlinear governing equations are obtained by introducing the von Kármán nonlinear displacement–strain relationship into the first-order shear deformation theory and dispersed via the differential quadrature (DQ) method. Combining the governing equation of the impactor’s motion, they are further parametrically solved by the Newmark-β method associated with the Newton–Raphson iterative process. The influence of different types of geometrical imperfections on the nonlinear thermomechanical low-velocity impact behaviors of GRC beams with varying weight fractions of GPLs, subjected to different initial impact velocities, are studied in detail.

## 1. Introduction

Geometric imperfections, commonly caused by manufacturing processes and environmental factors, play a critical role in influencing the mechanical behavior of structures, particularly in terms of the vibration, buckling, and linear and nonlinear dynamics [1,2,3,4,5]. Sheinman et al. [4] considered an isotropic beam subjected to a nonlinear elastic foundation with such imperfections and investigated its imperfection sensitivity. Similarly, Farokhi et al. [6] explored how geometric imperfections affect the nonlinear resonance behavior of a microbeam using the Timoshenko beam theory. To simulate various localized and global imperfections, Wadee [7] proposed a one-dimensional imperfection model and focused on the buckling and post-buckling behavior of the geometrically imperfect sandwich panels. Their research consistently demonstrated that structures are highly sensitive to geometric imperfections, underscoring the importance of studying these effects for accurate engineering design.

Beyond the isotropic homogeneous structures previously discussed, advanced high-strength and lightweight materials, along with high-performance composite structures, are increasingly being utilized in engineering and aerospace [8,9]. These include functionally graded materials (FGMs), fiber-reinforced composites, and carbon-based composites [10,11,12,13,14,15,16]. Due to the high surface area of nanoporous carbon materials, they have been widely utilized in myriad fields, including pharmaceutical [17] and mechanical applications [18].

As a promising material for enhancing the mechanical properties of composites, graphene nanoplatelets (GPL) are emerging as a nanofiller in the field of nanocomposites due to the ease and low cost of manufacturing [18]. Numerous theoretical analyses and experimental tests have demonstrated that dispersing GPLs in a polymer matrix can significantly improve its thermal and mechanical performance [19,20]. Yang and his team pioneered the concept of functionally graded (FG) GPL-reinforced composite (GRC) structures, investigating their bending, buckling, and vibration characteristics in depth [21,22,23]. Rahimi et al. [24,25] examined FG-GRC cylindrical shells and studied their three-dimensional free vibration and bending through a semi-analytical approach. Mao et al. [26,27,28,29] further investigated the linear and nonlinear behaviors of these intact FG-GRC structures subjected to complex loadings. Additionally, under various loading conditions, including moving loads [30], transverse excitation [31], and impact loads [32], many researchers have presented studies on the stability and vibration of FG-GRC structures. These studies consistently show that GPLs can significantly improve the stiffness of the GRC structures, with the degree of improvement largely determined by the GPL distribution pattern within the matrix.

Research on the nonlinear behavior of FG-GRC structures with geometric imperfections has shown that the shape and amplitude of these imperfections significantly influence the nonlinear vibration [2], buckling [33], and resonance [34]. Impact, a common load in engineering applications, can cause internal damage and even structural failure. Fan et al. [35,36], using a modified Hertz model, investigated the response of FG-GRC structures resting on a viscoelastic foundation, subjected to a low-velocity impact. Considering the combined axial and thermal loads, Dong et al. [37] employed an energy–balance and spring–mass model to predict the low-velocity impact response of FG-GRC cylindrical shells. Using a meshless approach, Selim et al. [38] studied the impact behavior of FG-GRC plates resting on Winkler–Pasternak elastic foundations. These studies underscore the importance of understanding how FG-GRC structures respond to impact to ensure their structural integrity.

Reviewing the public literature about the static and dynamic behaviors of the FG-GRC structures, it is found that the behaviors of the intact GRC structures have been comprehensively explored, but the low-velocity impact behavior of geometrically imperfect GRC structures has been less studied, especially under multi-physical fields. Only Zhang et al. [39] focused on the effect of different GPL distribution patterns on the low-velocity impact response of a geometrically imperfect GRC beam. The varying responses of a GRC beam with different types of imperfection have never been discussed, which is crucial for the application of the GRC structures.

This paper studies the low-velocity impact response of a GRC beam with varying geometrical imperfections, including the sine, global, and local imperfections to understand the different mechanical mechanisms of the low-velocity impact response of a GRC beam with varying geometrical imperfections. The thermal environment is also taken into consideration. The nonlinear thermomechanical response of geometrically imperfect GRC beams subjected to low-velocity impacts, incorporating the effects of temperature variations, varying weight fractions of GPLs, and initial impact velocities, is investigated. By employing a modified nonlinear Hertz contact law, along with the von Kármán nonlinear displacement–strain relationship and the first-order shear deformation theory, we aim to provide a comprehensive understanding of how these factors influence the dynamic response of GRC beams. The outcomes of this study offer significant insights into optimizing GRC beam designs for impact resilience in thermal environments.

## 2. Theoretical Models

**Figure 1:**
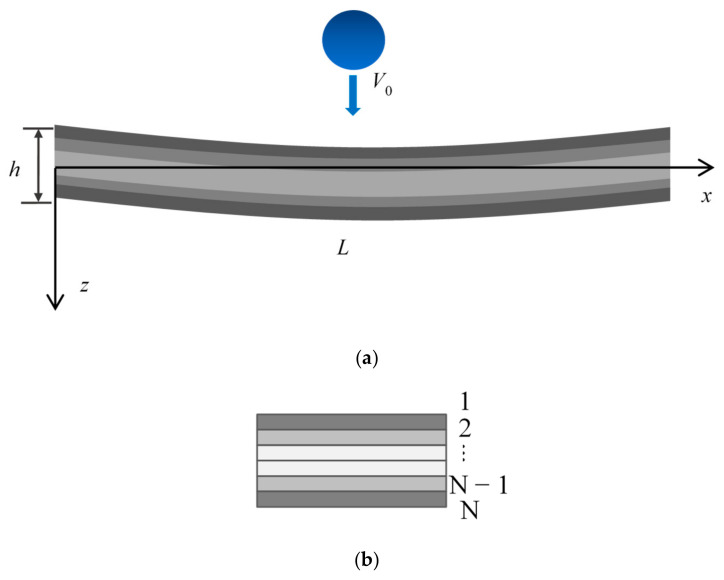
(**a**) *N*-layered geometrically imperfect GRC beam subjected to low-velocity impact and (**b**) the schematic diagram of the functionally graded distributed GPL along the thickness of the beam.

Figure 1 shows an *N*-layered geometrically imperfect GRC beam of length *L*, width *b*, and thickness *h*, with a uniform temperature variation $\Delta T=T-T_{0}$. To emphasize the coupled effect of the thermal environment and geometrical imperfections of the GRC beam, the material properties are considered as temperature-independent. A spherical impactor with mass *m_i_*, radius *R_i_*, and initial velocity *V*_0_ will impact the geometrically imperfect GRC beam at *x* = *x*_C_. The GPLs with length $l_{G}$, width $w_{G}$, and thickness $h_{G}$ are considered to disperse into the isotropic matrix along the thickness of the beam as a functionally graded pattern. In Figure 1b, the color represents the GPL volume fraction. As observed, the GPL volume fraction is symmetric along the middle layer and increases from the middle layers to the top layer, i.e., the FG-X pattern is considered. With the total weight fraction $f_{G}$, the GPL volume fraction $V_{G}^{\left(k\right)}$ (k=1,2,⋯,N) of the *k*-th layer in the GRC beam can be calculated by [40]

(1) $$V_{G}^{\left(k\right)}=\frac{2f_{G}}{f_{G}+\left(\rho_{G}/\rho_{M}\right)\left(1-f_{G}\right)}\frac{\left|2k-N-1\right|}{N},$$

where $\rho_{G}$ represents the mass density, *N* is the total number of the GRC sub-layers of the novel beam, and the subscripts “*G*” and “*M*” are, respectively, the GPLs and the matrix.

In this paper, we focus on the GPL reinforcements with no more than wt. 0.5%. The GPLs with such a low weight fraction tend to disperse into the epoxy with random orientation, and the Halpin–Tsai model [19] is appropriated to model the effective Young’s modulus $E_{C}^{\left(k\right)}$ of the *k*-th GRC layer:

(2) $$E_{C}^{\left(k\right)}=\frac{3\;\left(1+2\xi_{LW}\xi_{WH}\eta_{L}V_{G}^{\left(k\right)}\right)}{8\;\left(1-\eta_{L}V_{G}^{\left(k\right)}\right)}\times E_{M}+\frac{5\;\left(1+2\xi_{WH}\eta_{W}V_{G}^{\left(k\right)}\right)}{8\;\left(1+\eta_{W}V_{G}^{\left(k\right)}\right)}\times E_{M},$$

where

(3) $$\eta_{L}=\frac{Y_{G}/Y_{M}-1}{Y_{G}/Y_{M}+2\xi_{LW}\xi_{WH}},\;\eta_{W}=\frac{Y_{G}/Y_{M}-1}{Y_{G}/Y_{M}+2\xi_{WH}},$$

with

(4) $$\xi_{LW}=\frac{l_{G}}{w_{G}},\;\xi_{WH}=\frac{w_{G}}{h_{G}}.$$

According to the rule of mixture, the Poisson ratio $\nu_{C}^{\left(k\right)}$, mass density $\rho_{C}^{\left(k\right)}$, and thermal expansion coefficient $\alpha_{C}^{\left(k\right)}$ of the *k*-th GRC layer can be expressed as

(5) $$\rho_{C}^{\left(k\right)}=\rho_{G}V_{G}^{\left(k\right)}+\rho_{M}\left(1-V_{G}^{\left(k\right)}\right),$$

(6) $$\nu_{C}^{\left(k\right)}=\nu_{G}V_{G}^{\left(k\right)}+\nu_{M}\left(1-V_{G}^{\left(k\right)}\right),$$

and

(7) $$\alpha_{C}^{\left(k\right)}=\alpha_{G}V_{G}^{\left(k\right)}+\alpha_{M}\left(1-V_{G}^{\left(k\right)}\right).$$

**Figure 2:**
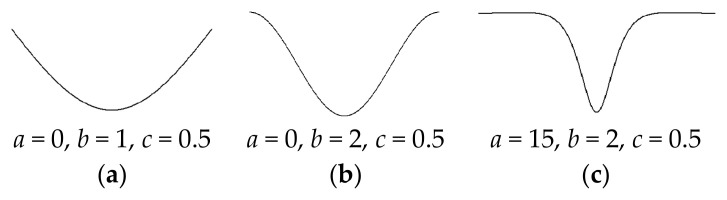
Types of imperfection: (**a**) sine, (**b**) global, and (**c**) local.

In realistic manufacturing, a mold is always used to product the main structures. The least stable localized buckling for the structure on a foundation is hard to avoid, which causes the initial geometric imperfections. The geometric imperfection $w^{*}\left(x\right)$ considered in this paper is simulated as the product of hyperbolic and trigonometric functions, which closely matches the least stable localized buckling for the structure on a foundation [7,41]:

(8) $$w^{*}\left(x\right)=A_{0}h\;\mathrm{sech}\left[a\left(x/L-c\right)\right]\;\cos\left[b\pi \left(x/L-c\right)\right],$$

where *h* is the thickness of the beam, *a*, *c*, and *b* are constants, and *A*_0_ is the dimensionless amplitude of the geometric imperfection. As seen in Figure 2, three different kinds of imperfections are analyzed in this paper. The sine imperfection has *a* = 0, *b* = 1, and *c* = 0.5. The global imperfection has *a* = 0, *b* = 2, and *c* = 0.5. The local imperfection has *a* = 15, *b* = 2, and *c* = 0.5.

To obtain the impact force $F_{C}\left(t\right)$ caused by the impactor, the Hertz contact theory is utilized [42,43,44], and the loading and unloading phases are distinguished by the maximum impact force $F_{\max}$ and the corresponding local contact indentation $\alpha_{\max}$.

As is known, the local contact indentation $\alpha \left(t\right)$ can be calculated by the displacement of the impactor $y_{i}\left(t\right)$ and that of the impacted point of the geometrically imperfect GRC beam $w_{C}\left(t\right)$:

(9) $$\alpha \left(t\right)=y_{i}(t)-w_{C}(t).$$

During the loading phase [43], it increases from zero to the maximum local contact indentation. The corresponding impacted force $F_{C}\left(t\right)$ can be yielded using the modified nonlinear Hertz contact reported by Yang and Sun [44]:

(10) $$F_{C}\left(t\right)=K_{C}\alpha^{3/2}\left(t\right),$$

where $K_{C}$ is the contact stiffness:

(11) $$K_{C}=\frac{4}{3}\sqrt{R_{i}}{\left(\frac{1-{\nu_{i}}^{2}}{E_{i}}+\frac{1-\nu_{C}^{\left(1\right)2}}{E_{C}^{\left(1\right)}}\right)}^{-1}.$$

The subscript “*i*” represents the impactor.

During the unloading phase, the local contact indentation $\alpha \left(t\right)$ decreases from the maximum local contact indentation to zero. The corresponding impacted force $F_{C}\left(t\right)$ can be expressed as [43]

(12) $$F_{C}\left(t\right)=F_{\max}{\left(\frac{\alpha \left(t\right)-\alpha_{0}}{\alpha_{\max}-\alpha_{0}}\right)}^{5/2},$$

with the permanent indentation $\alpha_{0}$ of the geometrically imperfect GRC beam. It is noted that the permanent indentation $\alpha_{0}=0$ as only elastic deformation is considered here.

## 3. Dynamic Equations

According to the first-order shear deformation theory and the initial geometrical imperfection $w^{*}\left(x\right)$, the axial and transverse displacements $\tilde{u}\left(x,z,t\right)$ and $\tilde{w}\;\left(x,z,t\right)$ of the geometrically imperfect GRC beam can be expressed as

(13) $$\tilde{u}\left(x,z,t\right)=u\left(x,t\right)+z\phi \left(x,t\right),\;\tilde{w}\;\left(x,z,t\right)=w\;\left(x,t\right)+w^{*}\left(x\right),$$

where *u*, *w*, and ϕ are the displacements of the axial, transverse, and rotary of the points in the mid-plane of the geometrically imperfect GRC beam, respectively.

The linear constitutive relation is utilized for the GRC layers:

(14) $$\sigma_{xx}^{\left(k\right)}=Q_{11}^{\left(k\right)}\left(\varepsilon_{xx}-\alpha_{C}^{\left(k\right)}\Delta T\right),\;\tau_{xz}^{\left(k\right)}=Q_{55}^{\left(k\right)}\gamma_{xz},$$

with the strain fields ($\varepsilon_{xx}$ and $\gamma_{xz}$) of the geometrically imperfect GRC beam, and

(15) $$Q_{11}^{\left(k\right)}=\frac{E_{C}^{\left(k\right)}}{1-\nu_{C}^{\left(k\right)2}},\;Q_{55}^{\left(k\right)}=\frac{E_{C}^{\left(k\right)}}{2\left(1+\nu_{C}^{\left(k\right)}\right)}.$$

The strain fields can be expressed according to the von Kármán nonlinear geometric relationship [45] as

(16) $$\varepsilon_{xx}=\frac{\partial u}{\partial x}+\frac{1}{2}{\left(\frac{\partial w}{\partial x}\right)}^{2}+z\frac{\partial \phi }{\partial x}+\frac{\partial w}{\partial x}\frac{dw^{*}}{dx},\;\gamma_{xz}=\frac{\partial w}{\partial x}+\phi .$$

In the framework of the Hamilton principle, the dynamic governing equations of the low-velocity impact behavior of the GRC beam under the coupled effect of thermal environment and geometrical imperfection can be obtained as

(17) $$\frac{\partial N_{x}}{\partial x}=I_{1}\frac{\partial^{2}u}{\partial t^{2}}+I_{2}\frac{\partial^{2}\phi }{\partial t^{2}},$$

(18) $$\frac{\partial }{\partial x}\left(N_{x}\frac{\partial w}{\partial x}+N_{x}\frac{\partial w^{*}}{\partial x}\right)+\frac{\partial Q}{\partial x}+F_{C}\left(t\right)\delta \left(x-x_{C}\right)=I_{1}\frac{\partial^{2}w}{\partial t^{2}},$$

(19) $$\frac{\partial M_{x}}{\partial x}-k_{s}Q_{x}=I_{2}\frac{\partial^{2}w}{\partial t^{2}}+I_{3}\frac{\partial^{2}\phi }{\partial t^{2}},$$

where the shear correction factor $k_{s}=5/6$, the inertia items *I*_1_, *I*_2_ and *I*_3_, and the resultant forces *N_x_*, *M_x_*, and *Q_x_* are given in Appendix A.

As the simply supported boundaries are considered, the displacements at the two ends of the geometrically imperfect GRC beam satisfy

(20) $$u=0,\;w=0,\;M_{x}=0.$$

Newton’s second law is utilized to obtain the governing equation of the impactor

(21) $$m_{i}{\ddot{y}}_{i}\left(t\right)+F_{C}\left(t\right)=0,$$

with the following initial conditions:

(22) $$y_{i}\left(0\right)=0,\;{\dot{y}}_{i}\left(0\right)=V_{0}.$$

We introduce the following nondimensional parameters:

(23) $$\left(U,W,W^{*}\right)=\frac{\left(u,w,w^{*}\right)}{h},\;\psi =\phi ,\;X=\frac{x}{L},\;\eta =\frac{L}{h},\;\tau =\frac{t}{L}\sqrt{\frac{A_{110}}{I_{10}}},\;\left(a_{11},a_{55},b_{11},d_{11}\right)=\left(\frac{A_{11}}{A_{110}},\frac{A_{55}}{A_{110}},\frac{B_{11}}{A_{110}h},\frac{D_{11}}{A_{110}h^{2}}\right),\;\left({\tilde{I}}_{1},{\tilde{I}}_{2},{\tilde{I}}_{3}\right)=\left(\frac{I_{1}}{I_{10}},\frac{I_{2}}{I_{10}h},\frac{I_{3}}{I_{10}h^{2}}\right),\;{\tilde{N}}^{T}=\frac{N^{T}}{A_{110}},\;{F_{C}}^{*}=\frac{F_{C}L}{A_{110}h},\;A_{110}=\frac{E_{M}}{1-{\nu_{M}}^{2}}h,\;I_{10}=\rho_{M}h.$$

Equations (17)–(19) can be rewritten as the nondimensional form:

(24) $$a_{11}\left[\frac{\partial^{2}U}{\partial X^{2}}+\frac{1}{\eta }\frac{\partial^{2}W}{\partial X^{2}}\left(\frac{\partial W}{\partial X}+\frac{dW^{*}}{dX}\right)+\frac{1}{\eta }\frac{\partial W}{\partial X}\frac{d^{2}W^{*}}{dX^{2}}\right]+b_{11}\frac{\partial^{2}\psi }{\partial X^{2}}={\tilde{I}}_{1}\frac{\partial^{2}U}{\partial \tau^{2}}+{\tilde{I}}_{2}\frac{\partial^{2}\psi }{\partial \tau^{2}},$$

(25) $$\left[a_{11}\left(\frac{1}{\eta }\frac{\partial^{2}U}{\partial X^{2}}+\frac{1}{\eta^{2}}\frac{\partial^{2}W}{\partial X^{2}}\frac{\partial W}{\partial X}+\frac{1}{\eta^{2}}\frac{\partial^{2}W}{\partial X^{2}}\frac{dW^{*}}{dX}+\frac{1}{\eta^{2}}\frac{\partial W}{\partial X}\frac{d^{2}W^{*}}{dX^{2}}\right)+\frac{b_{11}}{\eta }\frac{\partial^{2}\psi }{\partial X^{2}}\right]\left(\frac{\partial W}{\partial X}+\frac{dW^{*}}{dX}\right)\;+\left\{a_{11}\left[\frac{1}{\eta }\frac{\partial U}{\partial X}+\frac{1}{2\eta^{2}}{\left(\frac{\partial W}{\partial X}\right)}^{2}+\frac{1}{\eta^{2}}\frac{\partial W}{\partial X}\frac{dW^{*}}{dX}\right]+\frac{b_{11}}{\eta }\frac{\partial \psi }{\partial X}-{\tilde{N}}_{x}^{T}\right\}\left(\frac{\partial^{2}W}{\partial X^{2}}+\frac{d^{2}W^{*}}{dX^{2}}\right)\;+a_{55}\left(\frac{\partial^{2}W}{\partial X^{2}}+\eta \frac{\partial \psi }{\partial X}\right)+{F_{C}}^{*}\left(t\right)\delta \left(X-X_{C}\right)={\tilde{I}}_{1}\frac{\partial^{2}W}{\partial \tau^{2}},$$

(26) $$b_{11}\left[\frac{\partial^{2}U}{\partial X^{2}}+\frac{1}{\eta }\frac{\partial W}{\partial X}\frac{\partial^{2}W}{\partial X^{2}}+\frac{1}{\eta }\frac{\partial^{2}W}{\partial X^{2}}\frac{dW^{*}}{dX}+\frac{1}{\eta }\frac{\partial W}{\partial X}\frac{d^{2}W^{*}}{dX^{2}}\right]+d_{11}\frac{\partial^{2}\psi }{\partial X^{2}}-a_{55}\left(\eta \frac{\partial W}{\partial X}+\eta^{2}\psi \right)\;={\tilde{I}}_{2}\frac{\partial^{2}U}{\partial \tau^{2}}+{\tilde{I}}_{3}\frac{\partial^{2}\psi }{\partial \tau^{2}}.$$

## 4. Solution Procedure

To discuss the nonlinear low-velocity impact response of the GRC beam under the coupled effect of a thermal environment and geometrical imperfections, numerically, the differential quadrature (DQ) method [28,46] is employed alongside the Newmark-*β* method [30], in combination with the Newton–Raphson iterative process [47,48]. According to the DQ method, *U*, *W*, and ψ and their *r*-th derivatives with respect to $X_{i}$ in Equations (24)–(26) can be approximated as

(27) $$\left\{U,\;W,\;\psi \right\}=\sum_{m=1}^{n}l_{m}\left(X\right)\left\{U_{m},\;W_{m},\;\psi_{m}\right\},$$

(28) $${\left.\frac{\partial^{r}}{\partial X^{r}}\left\{U,\;W,\;\psi \right\}\right|}_{X=X_{i}}=\sum_{m=1}^{n}C_{im}^{\left(r\right)}\left\{U_{m},\;W_{m},\;\psi_{m}\right\},$$

with the Lagrange interpolation polynomial $l_{m}\left(X\right)$, the weighting coefficients $C_{im}^{\left(r\right)}$ of the *r*-th derivative at $X=X_{i}$,

(29) $$X_{i}=\frac{1}{2}\left[1-\cos\frac{\pi \left(i-1\right)}{n-1}\right]\;\left(i=1,\;2,\ldots ,n\right),$$

and the total number of nodes *n*.

Substituting Equations (27) and (28) into the nondimensional dynamic governing equations (Equations (24)–(26)) and the boundary conditions (Equation (20)), they can be dispersed as ordinary differential equations, as shown in Appendix B. Taking the stiffness-proportional damping matrix $C=\left(2\zeta /\omega_{l}\right)\;K_{L}$ [49,50] into consideration, the ordinary differential equations obtained from the DQ method can be rewritten as a matrix form:

(30) $$M\ddot{d}+C\dot{d}+\left(K_{L}+K_{NL}(d)\right)d=F+R,$$

with mass matrix M, structural linear stiffness matrix $K_{L}$, nonlinear stiffness matrix $K_{NL}$, modal damping factor ζ, and the corresponding natural frequency $\omega_{l}$, and

(31) $$d={\left\{{\left\{U_{i}\right\}}^{T},{\left\{W_{i}\right\}}^{T},{\left\{\psi_{i}\right\}}^{T}\right\}}^{T},\;i=1\cdots n,$$

(32) $$F=\left(\begin{array}{l} {\left\{0\right\}}_{n\times 1}\\ {\left\{{F_{C}}^{*}\left(\tau \right)\delta \left(X-X_{C}\right){|}_{X=X_{i}}\right\}}_{n\times 1}\\ {\left\{0\right\}}_{n\times 1} \end{array}\right),$$

(33) $$R=\left(\begin{array}{l} {\left\{0\right\}}_{n\times 1}\\ {\left\{{\tilde{N}}_{x}^{P}{\left.\frac{d^{2}W^{*}}{dx^{2}}\right|}_{X=X_{i}}\right\}}_{n\times 1}\\ {\left\{0\right\}}_{n\times 1} \end{array}\right).$$

To parametrically solve Equations (21) and (30) and obtain the low-velocity impact response, including the time-dependent impact force $F_{C}\left(t\right)$ and nonlinear deformations of the GRC beam under the coupled effect of the thermal environment and geometrical imperfections, the Newmark-β method [30] combined with the Newton–Raphson iterative method [47,48] is utilized with time step 10^−5^ s, as follows:

(1) Initially calculate: At the first time step, with an initial impact force *F_C_*_0_ and the predefined initial conditions $d_{0}$, ${\dot{d}}_{0}$, ${\ddot{d}}_{0}$, $y_{i0}$, and ${\dot{y}}_{i0}$, solve for the geometrically imperfect GRC beam $d_{1}$ and the displacements of the impactor $y_{i1}$ using Equations (21) and (30).
(2) Update the impact force: Using the displacements obtained in step 1, apply Equation (10) to calculate a new impact force *F_C_*_1_ and local contact indentation. If the indentation reaches the maximum value, recalculate *F_C_*_1_ using Equation (12). If *F_C_*_1_ is sufficiently close to *F_C_*_0_, proceed to step 3. Otherwise, take *F_C_*_1_ as the new initial impact force and repeat the displacement calculations.
(3) Iterate: Use the impact force and displacement obtained from the previous step as the initial conditions for the next time step. Continue iterating until the thermomechanical central displacement of the GRC beam converges and no longer changes significantly.

## 5. Results and Discussion

The nonlinear thermomechanical behaviors of a geometrically imperfect GRC beam subjected to low-velocity impact response are numerically analyzed, with a detailed discussion of the influences of the imperfect mode and amplitude *A*_0_, temperature variation ΔT, and the weight fraction *f_G_* of the GPLs, as well as the initial impact velocity *V*_0_ of the impactor on the time-dependent impact force and nonlinear thermomechanical central deflection of the novel beam.

The novel beam consists of epoxy with *E_M_* = 3.0 GPa, r_M_ = 1200 kg/m^3^, n_M_ = 0.34, and a_M_ = 60 × 10^−6^/K and GPLs with *E_G_* = 1010 GPa, r_G_ = 1060 kg/m^3^, n_G_ = 0.186, and a_G_ = 5 × 10^−6^/K [28,29]. The impactor is made of steel with *E_i_* = 207 GPa, r*_i_* = 7960 kg/m^3^, and n*_i_* = 0.3. Without a special statement, the geometric characteristics of the GRC beam and the GPLs are, respectively, *L* = 50 mm, *h* = 10 mm, and *A*_0_ = 0.02, and *l_G_* × *w*_G_ × *h_G_* = 2.5 mm × 1.5 mm × 1.5 nm. The total number *N* of the layered GRC beams with geometrical imperfections is selected as 12, and the damping factor ζ=0.1.

As the low-velocity impacts are defined as events that can occur in the range 1–10 m/s depending on the target stiffness, material properties, and the projectile mass and stiffness [51], to discuss the low-velocity impact behaviors of a geometrically imperfect GRC beam, the varying initial velocities *V*_0_ = 3 m/s, 5 m/s, and 7 m/s are selected. With distinct temperatures in the winter and summer, the temperature variations of 25 and 50 degrees centigrade are selected in our work. Since the GPLs are prone to agglomeration and poor dispersion at a high concentration, they may lead to considerably deteriorated mechanical properties and, hence, reduce the stiffness of the corresponding structures. Thus, only GPLs with a low concentration (wt.% < 1~3%) are widely used to enhance the structural mechanical behaviors. In this paper, we focus on the GPL reinforcements with no more than wt. 0.5%, i.e., *f_g_* = 0.1%, 0.3%, and 0.5%. Without a special statement, the impactor impacting *X_C_* = 0.5 has a radius *R_i_* = 5 mm, and the initial velocity *V*_0_ = 5 m/s. The thermal environment has a temperature variation ΔT=50 K, and the total weight fraction *f*_G_ = 0.3%.

**Figure 3:**
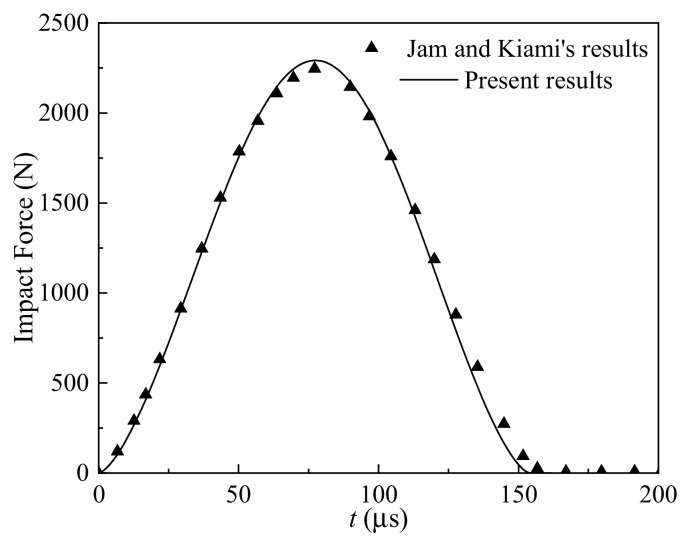
Comparison results: low-velocity impact force history of the intact CNTRC beam with FG-X pattern [52].

We studied the nonlinear thermomechanical behaviors of the geometrically imperfect GRC beam subjected to low-velocity impact response, and the convergence results and comparisons with the existing results are listed in Table 1 and presented in Figure 3 to ensure the correctness of the present results. The effect of the nodes’ number *n* on the maximum impact force *F*_c,max_ and thermomechanical central deflection *w*_mid,max_ of the geometrically imperfect GRC beam is presented. As seen, the GRC beams with sine and global imperfections have a higher convergence rate than a beam with a local imperfection. For the calculation correctness and efficiency, the node number n=13 is selected for the sine and global imperfections, and *n* = 37 for the local imperfection, in the following analyses. Figure 3 validates the correctness of the present results by comparing the low-velocity (*V*_0_ = 3 m/s) impact force history of the intact CNTRC beam with the FG-X pattern calculated by the present method with that reported by Jam and Kiani [52].

**Figure 4:**
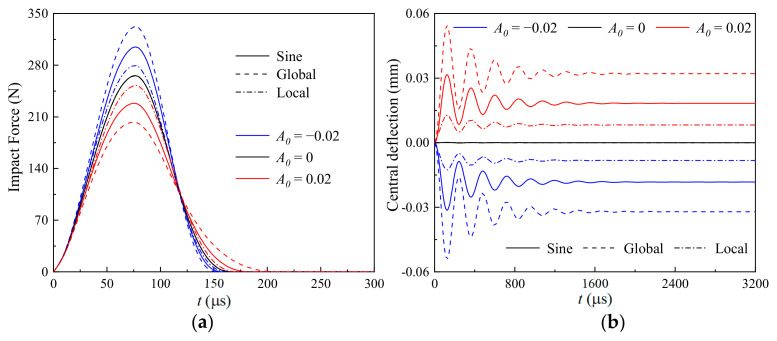
Influences of the imperfect mode and amplitude on the (**a**) time-dependent impact force and (**b**) nonlinear thermomechanical central deflection of a geometrically imperfect GRC beam.

Figure 4 shows the influences of the imperfect mode (sine, global, and local) and amplitude (*A*_0_ = −0.02, 0 and 0.02) on the (a) time-dependent impact force and (b) nonlinear thermomechanical central deflection of a geometrically imperfect GRC beam. The time-dependent impact force (as shown in Figure 4a) of a GRC beam with *A*_0_ < 0 is significantly greater than that with *A*_0_ > 0, and the impact force trends differ for the convex and concave imperfection amplitudes. This can be explained using Equations (9) and (10). The impact force during the impact process is determined by the difference between the displacements of the impactor $y_{i}\left(t\right)$ and the impacted point of the geometrically imperfect GRC beam $w_{C}\left(t\right)$, when the contact stiffness is given. The larger $w_{C}\left(t\right)$ is, the smaller the impact force is. Generated by the combined thermo-mechanical load and geometric imperfections, i.e., the force vector **R** in Equation (30), the beam with a concave imperfection amplitude (*A*_0_ > 0) has a positive pre-bending displacement, and the beam with a convex imperfection amplitude (*A*_0_ < 0) has a negative pre-bending displacement. This leads to *A_0_* > 0 having the largest $w_{C}\left(t\right)$, and $w_{C}\left(t\right)$. *A*_0_ < 0 is smaller than *A*_0_ = 0, which means the smallest impact force is for *A*_0_ > 0, and the largest impact force is for *A*_0_ < 0.

In addition, the GRC beam with a global imperfection has the highest thermomechanical central deflection, and the GRC beam with a local imperfection has the lowest thermomechanical central deflection, which is independent from the sign of *A*_0_. It is interesting that the influence of these three different imperfections on the impact force is highly dependent on the sign of *A*_0_. For *A*_0_ < 0, the globally imperfect GRC beam has the largest impact force, and the local imperfect one has the smallest impact force, while the influences are totally opposite when *A*_0_ > 0.

**Figure 5:**
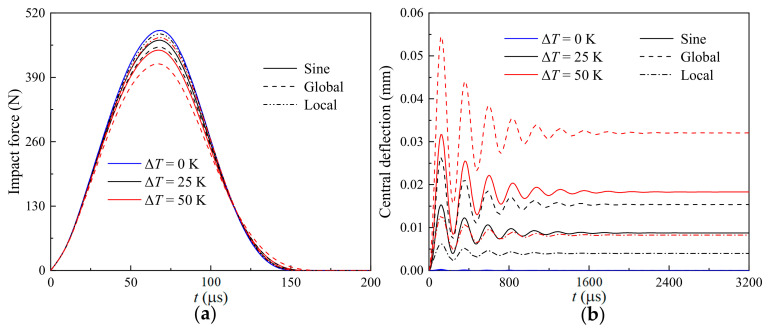
Effects of temperature variation on the (**a**) time-dependent impact force and (**b**) nonlinear thermomechanical central deflection of a GRC beam with different geometrical imperfections.

Figure 5 presents the effects of the distinct temperature variation (ΔT=0, 25 K, and 50 K) on the (a) time-dependent impact force and (b) nonlinear thermomechanical central deflection of a GRC beam with different geometrical imperfections (sine, global, and local). As the temperature variation increases, the force vector **R** increases and results in a decreasing impact force and an increasing thermomechanical central deflection. As *A_0_* = 0.02, i.e., *A*_0_ > 0, is considered here, the globally imperfect GRC beam has the smallest impact force and the largest thermomechanical central deflection, and the locally imperfect GRC beam has the largest impact force and the smallest thermomechanical central deflection. This is the same as the phenomena observed in Figure 4. It implies that the effect of the imperfect mode on the low-velocity impact response is independent from the varying temperature.

**Figure 6:**
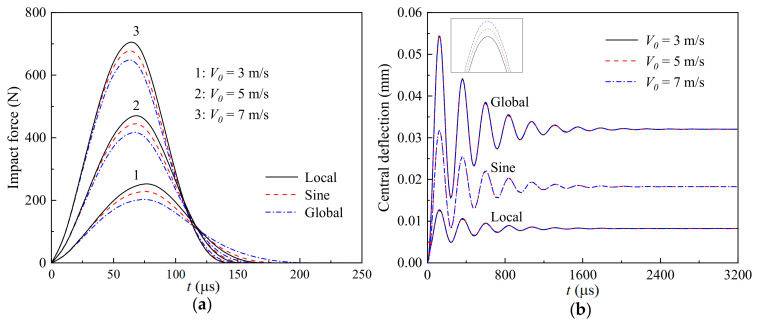
Effects of the initial impact velocity of the impactor on the (**a**) time-dependent impact force and (**b**) nonlinear thermomechanical central deflection of a GRC beam with different geometrical imperfections.

Figure 6 shows the effect of the initial impact velocity (*V*_0_ = 3 m/s, 5 m/s, and 7 m/s) of the impactor on the (a) time-dependent impact force and (b) nonlinear thermomechanical central deflection of a GRC beam with different geometrical imperfections (sine, global, and local). As predicted, the larger the initial impact velocity is, the larger the impact force and thermomechanical central deflection are. In addition, it can also be observed that the effect of the imperfect mode on the low-velocity impact response is independent from the initial impact velocity of the impactor.

**Figure 7:**
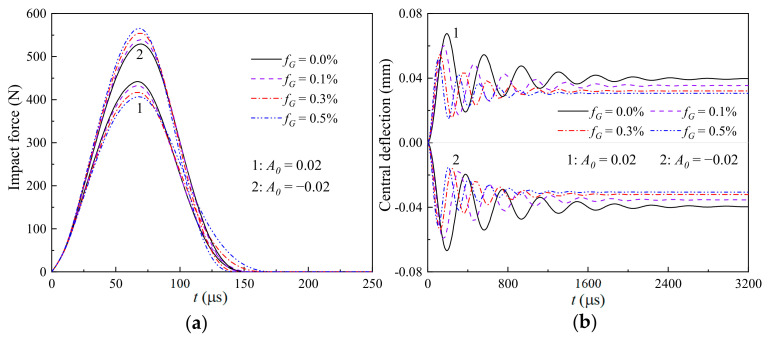
Effects of the weight fraction of GPLs and imperfect amplitude on the (**a**) time-dependent impact force and (**b**) nonlinear thermomechanical central deflection of a GRC beam with global imperfections.

**Figure 8:**
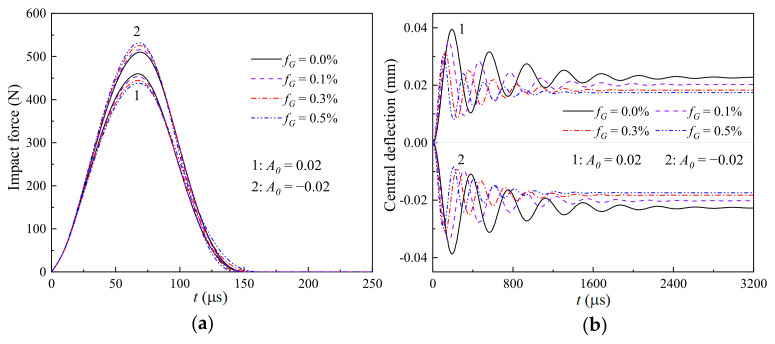
Effects of the weight fraction of GPLs and imperfect amplitude on the (**a**) time-dependent impact force and (**b**) nonlinear thermomechanical central deflection of a GRC beam with sine imperfections.

**Figure 9:**
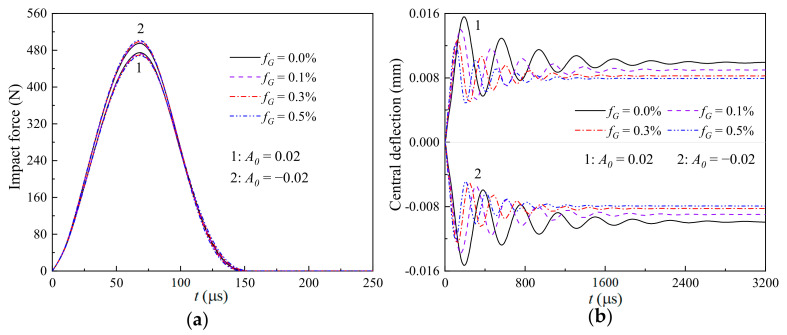
Effects of the weight fraction of GPLs and imperfect amplitude on the (**a**) time-dependent impact force and (**b**) nonlinear thermomechanical central deflection of a GRC beam with local imperfections.

Figure 7, Figure 8 and Figure 9 illustrate the effects of the weight fraction of the GPLs ($f_{G}=0.0\%$, 0.1%, 0.3%, and 0.5%) and the imperfect amplitude (*A*_0_ = −0.02 and 0.02) on the (a) time-dependent impact force and (b) nonlinear thermomechanical central deflection of the GRC beams with global, sine, and local imperfections, respectively. It is seen that the global case is the most sensitive to the varying weight fraction of the GPLs, and the local case is the least sensitive. Observing Figure 7b, Figure 8b and Figure 9b, the increasing weight fraction of the GPLs can significantly reduce the thermomechanical central deflections of the geometrically imperfect GRC beams, whether the imperfect amplitude is positive or negative. Meanwhile, the weight fraction of the GPLs has a different effect on the impact force of the geometrically imperfect GRC beam with different imperfect amplitudes.

For the beams with the same GPL weight fraction but different imperfection characteristics, i.e., the contact stiffness is given, the impact force during the impacting process is determined by the difference between the displacements of the impactor $y_{i}\left(t\right)$ and the impacted point of the geometrically imperfect GRC beam $w_{C}\left(t\right)$. Because of the pre-bending caused by the combined thermo-mechanical load and geometric imperfections, R in Equation (30), *A*_0_ > 0 has a smaller impact force than *A*_0_ < 0, as presented in Figure 4. In addition, the increasing weight fraction of the GPLs can increase the impact force for the GRC beam with convex characteristics (*A*_0_ < 0) but decrease the impact force for the GRC beam with concave characteristics (*A*_0_ > 0), as observed from Figure 7a, Figure 8a and Figure 9a. In general, the increasing weight fraction of the GPLs implies an increasing contact stiffness, and combined with the negative pre-bending, it will further increase the impact force for *A*_0_ < 0. However, the increasing weight fraction of the GPLs decreases the impact force for the GRC beam with *A*_0_ > 0. This implies that the pre-bending caused by the coupled effect of the thermal environment and the geometrical imperfections plays a dominant role in the nonlinear thermomechanical low-velocity impact behaviors of a geometrically imperfect GRC beam.

## 6. Conclusions

The nonlinear low-velocity impact behaviors of a GRC beam under the coupled effect of the thermal environment and geometrical imperfections are investigated. The impact force is calculated by the modified nonlinear Hertz contact law. In the framework of the von Kármán nonlinear displacement–strain relationship, first-order shear deformation beam theory, and Hamilton’s principle, the nonlinear dynamic equations are firstly deduced, then dispersed by the DQ method, and parametrically solved by the Newmark-β method combined with the Newton–Raphson iterative method. The influences of the imperfect mode and amplitude, temperature variation, weight fraction of GPLs, and the initial impact velocity of the impactor on the nonlinear thermomechanical low-velocity impact responses of the geometrically imperfect GRC beam are examined. The main results can be concluded as follows:

(1) The GRC beams with sine and global imperfections have a higher convergence rate on the node number than a beam with a local imperfection. The global case is the most sensitive to the varying weight fraction of the GPLs, and the local case has the least sensitivity.
(2) Among these three different imperfections, the global imperfection always has the highest thermomechanical central deflection, which is independent from the sign of the imperfect amplitude, while their impact forces are closely dependent on the sign of the imperfect amplitude.
(3) The increasing weight fraction of the GPLs can increase the impact force of the GRC beam with convex characteristics but decrease the impact force of the GRC beam with concave characteristics.

## Figures and Tables

**Table 1:** Convergence results: effect of nodes’ number *n* on the maximum impact force and thermomechanical central deflection of a geometrically imperfect GRC beam.

*n*	*F*_c,max_			*w*_mid,max_		
	Sine	Global	Local	Sine	Global	Local
13	443.27	415.29	333.52	0.0318	0.0546	0.1267
19	443.38	415.4	443.63	0.0317	0.0545	0.0336
25	443.44	415.46	464.91	0.0316	0.0544	0.0166
31	443.48	415.46	468.94	0.0316	0.0544	0.0134
37	443.5	415.5	469.48	0.0316	0.0544	0.0129
43	443.52	415.53	469.61	0.0316	0.0544	0.0128
49	443.53	415.54	469.64	0.0316	0.0544	0.0128

## Data Availability

The original contributions presented in the study are included in the article, further inquiries can be directed to the corresponding authors.

## Appendix A

The inertia items, *I*_1_, *I*_2_, and *I*_3_, and the resultant forces, *N_x_*, *M_x_,* and *Q_x_*, in Equations (17)–(19) are calculated by

(A1) $$\left(\begin{array}{l} I_{1}\\ I_{2}\\ I_{3} \end{array}\right)=\sum_{k=1}^{N}\int_{h/2-k\Delta h}^{h/2-\left(k-1\right)\Delta h}\rho_{C}^{\left(k\right)}\left(\begin{array}{l} 1\\ z\\ z^{2} \end{array}\right)\;dz,$$

(A2) $$N_{x}=A_{11}\left[\frac{\partial u}{\partial x}+\frac{1}{2}{\left(\frac{\partial w}{\partial x}\right)}^{2}+\frac{\partial w}{\partial x}\frac{\partial w^{*}}{\partial x}\right]+B_{11}\frac{\partial \phi }{\partial x}-N_{x}^{T},$$

(A3) $$M_{x}=B_{11}\left[\frac{\partial u}{\partial x}+\frac{1}{2}{\left(\frac{\partial w}{\partial x}\right)}^{2}+\frac{\partial w}{\partial x}\frac{\partial w^{*}}{\partial x}\right]+D_{11}\frac{\partial \phi }{\partial x}-M_{x}^{T},$$

(A4) $$Q_{x}=A_{55}\left(\frac{\partial u}{\partial x}+\phi \right),$$

with the stiffness components

(A5) $$\left(\begin{array}{l} A_{11}\\ B_{11}\\ D_{11} \end{array}\right)=\sum_{k=1}^{N}\int_{h/2-k\Delta h}^{h/2-\left(k-1\right)\Delta h}Q_{11}^{\left(k\right)}\left(\begin{array}{l} 1\\ z\\ z^{2} \end{array}\right)dz,\;A_{55}=\sum_{k=1}^{N}\int_{h/2-k\Delta h}^{h/2-\left(k-1\right)\Delta h}Q_{55}^{\left(k\right)}k_{s}dz,$$

and the thermal related loads

(A6) $$\left(\begin{array}{l} N_{x}^{T}\\ M_{x}^{T} \end{array}\right)=\sum_{k=1}^{N}\int_{h/2-k\Delta h}^{h/2-\left(k-1\right)\Delta h}Q_{11}^{\left(k\right)}\alpha_{c}^{\left(k\right)}\left(\begin{array}{l} 1\\ z \end{array}\right)\;dz,$$

## Appendix B

With the DQ method, the dynamic governing equations (Equations (24)–(26)) for the nonlinear low-velocity impact response of a geometrically imperfect GRC beam, in nondimensional form, can be expressed as the following ordinary differential equations:

(A7) $$a_{11}\sum_{m=1}^{n}C_{im}^{\left(2\right)}U_{m}+\frac{a_{11}}{\eta }\left[\sum_{m=1}^{n}C_{im}^{\left(1\right)}W_{m}\sum_{m=1}^{n}C_{im}^{\left(2\right)}W_{m}+{\left.\frac{dW^{*}}{dx}\right|}_{X=X_{i}}\sum_{m=1}^{n}C_{im}^{\left(2\right)}W_{m}\;\right.\left.+{\left.\frac{d^{2}W^{*}}{dx^{2}}\right|}_{X=X_{i}}\sum_{m=1}^{n}C_{im}^{\left(1\right)}W_{m}\right]+b_{11}\sum_{m=1}^{n}C_{im}^{\left(2\right)}\psi_{m}={\tilde{I}}_{1}{\ddot{U}}_{i}+{\tilde{I}}_{2}{\ddot{\psi }}_{i},$$

(A8) $$\left[\frac{a_{11}}{\eta }\sum_{m=1}^{n}C_{im}^{\left(2\right)}U_{m}+\frac{a_{11}}{\eta^{2}}\left(\sum_{m=1}^{n}C_{im}^{\left(1\right)}W_{m}\sum_{m=1}^{n}C_{im}^{\left(2\right)}W_{m}+{\left.\frac{dW^{*}}{dx}\right|}_{X=X_{i}}\sum_{m=1}^{n}C_{im}^{\left(2\right)}W_{m}+{\left.\frac{d^{2}W^{*}}{dx^{2}}\right|}_{X=X_{i}}\sum_{m=1}^{n}C_{im}^{\left(1\right)}W_{m}\right)\;\right.+\left.\frac{b_{11}}{\eta }\sum_{m=1}^{n}C_{im}^{\left(2\right)}\psi_{m}\right]\left(\sum_{m=1}^{n}C_{im}^{\left(1\right)}W_{m}+{\left.\frac{dW^{*}}{dx}\right|}_{X=X_{i}}\right)+\left(\frac{a_{11}}{\eta }\sum_{m=1}^{n}C_{im}^{\left(1\right)}U_{m}\right.+\frac{a_{11}}{\eta^{2}}{\left.\frac{dW^{*}}{dx}\right|}_{X=X_{i}}\sum_{m=1}^{n}C_{im}^{\left(1\right)}W_{m}\;+\frac{a_{11}}{2\eta^{2}}\sum_{m=1}^{n}C_{im}^{\left(1\right)}W_{m}\sum_{m=1}^{n}C_{im}^{\left(1\right)}W_{m}+\left.\frac{b_{11}}{\eta^{2}}\sum_{m=1}^{n}C_{im}^{\left(1\right)}\psi_{m}-{\left.{\tilde{N}}_{x}^{T}\right|}_{X=X_{i}}\right)\left(\sum_{m=1}^{n}C_{im}^{\left(2\right)}W_{m}\right.\left.+{\left.\frac{d^{2}W^{*}}{dx^{2}}\right|}_{X=X_{i}}\right)\;+a_{55}\left(\sum_{m=1}^{n}C_{im}^{\left(2\right)}W_{m}+\eta \sum_{m=1}^{n}C_{im}^{\left(1\right)}\psi_{m}\right)={\tilde{I}}_{1}{\ddot{W}}_{i}-{F_{C}}^{*}\left(\tau \right)\delta {\left(X-X_{C}\right)|}_{X=X_{i}}$$

(A9) $$b_{11}\sum_{m=1}^{n}C_{im}^{\left(2\right)}U_{m}+\frac{b_{11}}{\eta }\left(\sum_{m=1}^{n}C_{im}^{\left(1\right)}W_{m}\sum_{m=1}^{n}C_{im}^{\left(2\right)}W_{m}+{\left.\frac{dW^{*}}{dx}\right|}_{X=X_{i}}\sum_{m=1}^{n}C_{im}^{\left(2\right)}W_{m}+{\left.\frac{d^{2}W^{*}}{dx^{2}}\right|}_{X=X_{i}}\sum_{m=1}^{n}C_{im}^{\left(1\right)}W_{m}\right)\;+d_{11}\sum_{m=1}^{n}C_{im}^{\left(2\right)}\psi_{m}-a_{55}\left(\eta \sum_{m=1}^{n}C_{im}^{\left(1\right)}W_{m}+\eta^{2}\psi_{i}\right)={\tilde{I}}_{2}{\ddot{U}}_{i}+{\tilde{I}}_{3}{\ddot{\psi }}_{i}.$$

Similarly, the boundaries in Equation (20) can be dispersed as

(A10) $$U_{1}=0,\;W_{1}=0,\;b_{11}\left[\sum_{m=1}^{n}C_{1m}^{\left(1\right)}U_{m}+\frac{1}{2\eta }{\left(\sum_{m=1}^{n}C_{1m}^{\left(1\right)}W_{m}\right)}^{2}+\frac{1}{\eta }{\left.\frac{dW^{*}}{dx}\right|}_{X=X_{1}}\sum_{m=1}^{n}C_{1m}^{\left(1\right)}W_{m}\right]\;+d_{11}\sum_{m=1}^{n}C_{1m}^{\left(1\right)}\Psi_{m}-{\left.{M_{x}}^{T}\right|}_{X=X_{1}}=0,$$

and

(A11) $$U_{n}=0,\;W_{n}=0,\;b_{11}\left[\sum_{m=1}^{n}C_{nm}^{\left(1\right)}U_{m}+\frac{1}{2\eta }{\left(\sum_{m=1}^{n}C_{nm}^{\left(1\right)}W_{m}\right)}^{2}+\frac{1}{\eta }{\left.\frac{dW^{*}}{dx}\right|}_{X=X_{n}}\sum_{m=1}^{n}C_{nm}^{\left(1\right)}W_{m}\right]\;+d_{11}\sum_{m=1}^{n}C_{nm}^{\left(1\right)}\Psi_{m}-{\left.{M_{x}}^{T}\right|}_{X=X_{n}}=0.$$
